# A Novel Approach to Predict the Growth of *Staphylococcus aureus* on Rice Cake

**DOI:** 10.3389/fmicb.2017.01140

**Published:** 2017-06-22

**Authors:** Jun Wang, Shige Koseki, Mi-Ja Chung, Deog-Hwan Oh

**Affiliations:** ^1^College of Food Science and Engineering, Qingdao Agricultural UniversityQingdao, China; ^2^Research Faculty of Agriculture, Hokkaido UniversitySapporo, Japan; ^3^Department of Food Science and Nutrition, College of Health, Welfare and Education, Gwangju UniversityGwangju, South Korea; ^4^Department of Food Science and Biotechnology, Institute of Bioscience and Biotechnology, Kangwon National UniversityChuncheon, South Korea

**Keywords:** *Staphylococcus aureus*, predictive microbiology, rice cake, growth model, probability model

## Abstract

This study aimed to investigate the growth kinetics of *Staphylococcus aureus* on rice cake and to determine the shelf life based on the probability model of the increase in *S. aureus* contamination on rice cake. Secondary models were developed based on the growth parameters derived from the Baranyi model at constant temperatures (15, 25, 35, and 45°C). External validation was then conducted using additional data under experimental conditions not used in development of the models to verify the performance and reliability of the developed model through different goodness-of-fit indices. Furthermore, the growth of *S. aureus* on rice cake under dynamic temperature was obtained with the root mean square error (RMSE) of 0.218 and the 90.9% acceptable prediction rate. In addition, probability models of the 1-, 2-, 3-, and 4-log increases of *S. aureus* on rice cake were also developed from the data, which could provide the probability and the time to a certain log increase. The results of validation demonstrated that the developed predictive model and the obtained growth parameters could be used for evaluating the growth behavior of *S. aureus* on rice cake under different conditions, and qualified to supply sufficient information for microbiological risk assessment studies of *S. aureus* on rice cake in Korea.

## Introduction

Rice cake is one of the most popular Korean traditional foods. Many types of rice cake can be prepared via the use of different ingredients and different manufacturing processes. *Gyungdan*, a ball-shaped rice cake filled with delicious, sweet red bean paste and rolled in sesame seeds, is the most common variety of “tteok.” The major ingredients of rice cakes are rice flour and water, which causes rapid starch retrogradation during storage (Riva et al., 2000; Wu et al., 2009). Korean traditional rice cakes are generally packaged with linear low-density polyethylene after steaming and cooling at room temperature, and then distributed throughout markets (Lee et al., 2011). Oh et al. (2007) reported that 19.3% of the rice cakes with filling in Korea were contaminated with *Staphylococcus aureus*. The most important bacteria causing food poisoning in Korea are *Salmonella* spp., *Vibrio* spp., and *S. aureus*. These bacteria account for 85–90% of the outbreaks and cases of illness in Korea (Park et al., 2001). In addition, *Salmonella* spp., *S. aureus*, and *Bacillus cereus* are the major microbiological hazards of cereal grains and related products (FDA, 2003).

In the past decades, foodborne illness has been a serious public health issue in both developed and developing countries. *S. aureus* is able to produce a variety of toxins and many disease syndromes, and many detection method of *S. aureus* were developed and reported (Dabrowski and Medrala, 2004; Zhao et al., 2014; Li et al., 2016). In the United States, total 17 foodborne outbreaks caused by *Staphylococcus* enterotoxin and 566 outbreak-associated illnesses were reported to the CDC in 2014 (CDC, 2016). In Korea, 22.76% of the 10,676 foodborne illnesses from 1981 to 1995 caused by bacteria were associated with *S. aureus* strains, while 10.8% of the 33,353 patients suffering from food poisoning from 2001 to 2005 had illness related to enterotoxins produced by *S. aureus* (Lee et al., 2001; Yoon et al., 2011). Risk related to *S. aureus* and staphylococcal enterotoxins in fluid milk were estimated and demonstrated from the consumption of milk (Ding et al., 2016). It is necessary to estimate the effects of environmental factors on the growth and survival of *S. aureus* on rice cake, as it is the most popular traditional food in Korea.

The methodologies of primary and secondary modeling approaches and validation indices are based on well-established in many publications. But the ways to develop the growth probability model and the kinetic models under fluctuating temperature indicating the different log-increase probability at different temperatures and including as a dependent variable in the logistic models, respectively, are rarely reported. The objective of the present study was to develop a novel approach to predict the growth kinetics of *S. aureus* on rice cake under various environmental conditions. The Baranyi and Roberts model and Ratkowsky square root equation were selected to develop the primary and secondary models to evaluate the growth of *S. aureus* on rice cake under 15, 25, 35, and 45°C. In addition, the kinetic models under fluctuating temperature for the growth of *S. aureus* on rice cake was developed along with a probability model of 1-, 2-, 3-, and 4-log increase were also developed based on the *S. aureus* growth data on rice cakes to identify the shelf life.

## Materials and methods

### Preparation of strains

*Staphylococcus aureus* strains ATCC 12598, ATCC 25923, and ATCC 12600, obtained from the department of Food Science, University of Georgia, were used in this study. All strains were maintained at −70°C in tryptic soy broth (TSB, Difco, Sparks, MD, USA) containing 0.6% yeast extract (YE, Difco, USA) and 20% glycerol. For experimental purposes, the stock cultures of the strains were activated in tryptic soy broth (TSB; Difco, Detroit, MI, USA) at 35°C for 24 h in order to reach to the stationary phase. The bacteria were harvested by centrifugation (3,000 × g) at 4°C for 10 min, and then washed twice using 0.1% (w/v) sterile peptone water (PW; Difco, USA). The bacterial suspensions were mixed at equal concentrations to obtain a mixture with the final population level of approximately 5 log cfu/mL.

### Preparation and inoculation of samples

Gyungdan, a ball-shaped rice cake filled with sweet red bean paste and rolled in sesame seeds, were purchased from a local supermarket in Chuncheon, Korea. The pH and water activity of rice cake was approximately 6.14 and 0.975, respectively. The samples were cut in half using a sterile knife and placed on sterile aluminum foil in a lamella flow hood. The rice cakes were divided into 10 g samples with a balance to prepare for inoculation. For inoculation, 0.1 mL of the mixed culture (5 log cfu/mL) was applied to the samples by depositing droplets with a micropipettor. This procedure resulted in an initial pathogen inocula level of approximately 3 log cfu/g.

### Growth experiments of *S. aureus*

The inoculated samples were packaged with polyethylene and transferred into the growth chamber (BF-600GC, BioFree, Seoul, Korea). Uninoculated samples were used as a control. For the purpose of model development, the inoculated samples were stored at 5, 15, 25, 35, and 45°C at two different levels (50 and 80%) of relative humidity until they reached the stationary phase. As for model validation, the experiments were conducted at 10, 20, 30, and 40°C within the region of interpolation of the model. Sampling was generally carried out for enumeration based on the designed intervals, depending on the different incubation temperatures. Two samples were tested at each time interval. The growth studies were conducted in triplicate for each combination of conditions. Experimental period was set approximately 25 h at 35 and 45°C, 30 h at 30 and 40°C, and approximately 50 h at 20 and 25°C. However, longer periods were selected for the experiment at 5, 10, and 15°C.

### Enumeration of *S. aureus*

At each sampling time, the 10 g rice cake samples to be tested were taken from the growth chamber and put into a sterile 400-mL stomaching bag with a filter (Nasco Whirl-Pak, Janesville, WI, USA) then mixed with 90 mL of 0.1% sterilized PW. Samples were then pummeled in a Seward Stomacher (400 Circulator, Seward, London, UK) at 200 rpm for 2 min. After homogenization, 1 mL of the sample suspension containing *S. aureus* was added into 9 mL 0.1% sterilized PW, and serial 10-fold dilutions were performed using 0.1% sterilized PW. Subsequently, 0.1 mL of the obtained samples or dilutions was plated in duplicate on Baird-Parker Agar (Difco Co., USA). After incubation of the plates at 35°C for 24 h, colonies were counted and the data were expressed as a logarithm of the population (log cfu/g). Means of cell populations from each treatment were calculated from three replications.

### Kinetic growth model development

#### Primary modeling

Experimental data of *S. aureus* growth on the rice cake samples were collected for each designed trial with the different combinations of temperature and relative humidity. The specific growth rate (μ, 1/h) and lag time (LT, h) for each growth curve were generated by fitting the raw data into the Baranyi and Roberts model (Baranyi and Roberts, 1994). Analysis was carried out with R software (A language and environment for statistical computing. R Foundation for Statistical Computing, Vienna, Austria. ISBN 3-900051-07-0, URL http://www.R-project.org/). An R-software package *nlstools* was used to fit the Baranyi and Roberts model.

#### Secondary modeling

The Ratkowsky's square root model (Equation 1; Ratkowsky et al., 1982; Oscar, 2002) was used to develop secondary models for the μ, obtained from different primary models by non-linear regression using the Origin 8.5 software package (OriginLab corporation, Northampton, MA 01060, USA) based upon the generated growth parameters.

(1) $$\sqrt{\mu }=a(T-T_{min})$$

where μ is specific growth rate (1/h), *T* is the temperature (°C), *T*_*min*_ is the minimum temperature (°C) required for growth, and *a* is the regression coefficient.

#### Differential secondary modeling

*Staphylococcus aureus* growth under fluctuating temperature.

In order to simulate the growth of *S. aureus* on rice cake, the Baranyi and Roberts (1994) was implemented as follows:

(2) $$\frac{dq}{dt}=\mu q,q(0)=q_{0}$$

(3) $$\frac{dN}{dt}=\frac{q}{1+q}\mu \left(1-\frac{N}{N_{max}}\right)N,N(0)=N_{0}$$

where *N* denotes the bacterial cell concentration (CFU/g) at time *t, q* is a dimensionless quantity related to the physiological state of the cells, μ is the maximum specific growth rate (1/h), and *N*_max_ represents the maximum population density of the bacteria (CFU/g). The *q*_0_ and *N*_0_ represents the initial value of the *q* and *N*, respectively. For the initial value of *q*_0_ which is a measure of the initial physiological state of the cells, a geometric mean value for the physiological state parameter α_0_ was estimated from the constant temperature experimental data. It should be noted that the relationship between lag time (λ) and α_0_ could be shown as follows:

(4) $$\mu \times \text{LT}=\ln\left(1+\frac{1}{q_{0}}\right)=-\text{ln}(\alpha_{0})$$

The models for μ (Equation 1) along with *N*_max_ and *q*_0_ were substituted into Equations (2, 3), and the temperature allowed to be dependent on time. The system was solved numerically by the fourth-order Runge-Kutta method as a means of obtaining predictions of bacterial concentration using statistical software R (deSolve package). The temperature profile was recorded continuously using a data logger with an interval of 10 s and set to stimulate real life conditions during storage and transportation.

### Growth probability model development

For each growth probability model development, each sampling point was scored with values of 0 and 1 to indicate whether or not to obtain 1-, 2-, 3-, and 4-log increases of *S. aureus* growth, respectively. The probability of a 1-, 2-, 3-, and 4-log increases of *S. aureus* from the growth data on rice cakes was collected and fitted to a logistic regression model using R software (A language and environment for statistical computing. R Foundation for Statistical Computing, Vienna, Austria. ISBN 3-900051-07-0, URL http://www.R-project.org/). The analyses were made with the glm() function that fits generalized linear models in R-software package STATS. The initial model for fitting had the following form after a minor modification according to the approach described by Ratkowsky and Ross (1995), Koseki et al. (2009), and Agresti (2007):

(5) $$\text{Logit}\left(P\right)=\alpha_{0}+\alpha_{1}\text{Temp}+\alpha_{2}\;\cdot \text{ln}(\text{Time})$$

where *P* is the probability of an arbitrary log-increase based on *S. aureus* growth, $\text{Logit}\left(P\right)=\text{ln}\left(\frac{P}{1-P}\right)$, α_0_−α_2_ are the coefficients to be estimated, Temp is the storage temperature, and Time is the time at which the increase of *S. aureus* growth reached the set values.

To evaluate the goodness-of-fit the developed model, the maximum rescaled *R*-square statistic, the Hosmer-Lemeshow goodness-of-fit statistic, and the receiver operating characteristic (ROC) curve were used (Agresti, 2007). The maximum rescaled *R*^2^ for use with binomial error was proposed as a generalization of the coefficient of determination *R* that is commonly used in regression applications involving normally distributed error (Nagelkerke, 1991; Tienungoon et al., 2000). The Hosmer-Lemeshow goodness-of-fit statistic, which involves grouping objects into a contingency table and calculating a Pearson chi-square statistic, was proposed as a means of estimating goodness of fit (Tienungoon et al., 2000). Small values of the statistic (large *P*-values) indicate a good fit of the model to the data. The area under the ROC curve, *c*, is a measure of discrimination, obtained from a plot of sensitivity, i.e., the proportion of observed events that were correctly predicted to be events, against the complement of specificity, i.e., the proportion of nonevents that were correctly predicted to be nonevents. The closer the value of *c* is to 1, the greater is the discrimination. In epidemiological studies, a *c* value of >0.7 is considered acceptable discrimination, a *c* value of >0.8 as good discrimination, and a *c* value of >0.9 as excellent discrimination (Lemeshow and Jean-Roger, 1994).

### Validation of the predictive models

The performance and reliability of the developed models should be validated before actual application. The validation step was conducted using experimental data which was not used for model development. In the validation step, several statistical indices, such as bias factor (*B*_*f*_, Equation 6), accuracy factor (*A*_*f*_, Equation 7), the root mean square error (RMSE, Equation 8; McKellar and Lu, 2004) and %standard error of prediction (%SEP, Equation 9; Garcia-Gimeno et al., 2005) were employed as follows:

(6) $$B_{f}=10^{\left(\sum_{i=1}^{n}\log(\mu_{observed}/\mu_{predicted})/n\right)}$$

(7) $$A_{f}=10^{\left(\sum_{i=1}^{n}\left|\log(\mu_{predicted}/\mu_{observed})\right|/n\right)}$$

(8) $$RMSE=\sqrt{\frac{\sum {\left(\mu_{observed}-\mu_{predicted}\right)}^{2}}{n}}$$

(9) $$\%SEP=\frac{100}{\text{Average}\left(\mu_{observed}\right)}\sqrt{\frac{\sum {\left(\mu_{observed}-\mu_{predicted}\right)}^{2}}{n}}$$

where *n* is the number of observations, μ_*observed*_ is the observed value, and μ_*predicted*_ is the predicted value.

Differential secondary model performance was evaluated using the RMSE. In addition, the acceptable prediction zone method was used for evaluation of the model performance (Oscar, 2005). The prediction errors or relative errors (REs) for the individual fitted cases were calculated according to the following equation:

(10) $$\text{RE}=\frac{\text{Predicted }-\text{ Observed}}{\text{Predicted}}$$

The model gives fail-safe predictions when the RE is <0, while fail-dangerous predictions at RE values >0. The proportion of RE (pRE) in the acceptable prediction zone of RE −0.6 to 0.3, wherein the proportion of ≥0.70 indicated an acceptable model, was used to evaluate the performance of the obtained model.

### Statistical analysis

All the data from the three replicates were expressed as log colony forming units per gram (log CFU/g) and used together for model development. Statistical analysis was performed using IBM SPSS statistics 20 (IBM Corporation, New York, USA). Data were expressed as means ± standard deviation (SD). All data were analyzed using Analysis of variance (ANOVA). Statistical significance of growth parameters was analyzed by *t*-test at a significance level of 0.05. Values of validation indices were calculated in a Microsoft Excel spreadsheet.

## Results and discussion

### Growth of *S. aureus* on rice cake at different temperatures

**Figure 1:**
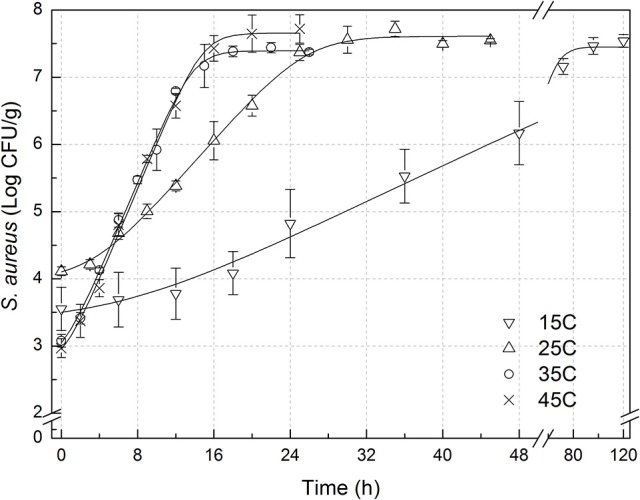
Observed growth data and fitted primary models of *Staphylococcus aureus* on rice cake at 15, 25, 35, and 45°C.

**Figure 2:**
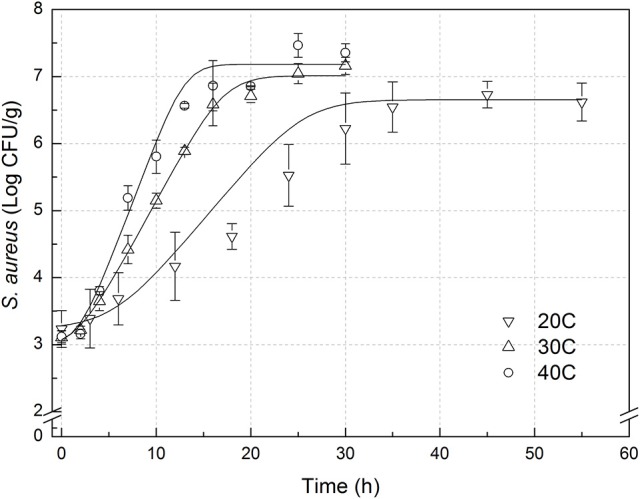
Observed growth data and fitted primary models of *Staphylococcus aureus* on rice cake at 20, 30, and 40°C.

**Figure 3:**
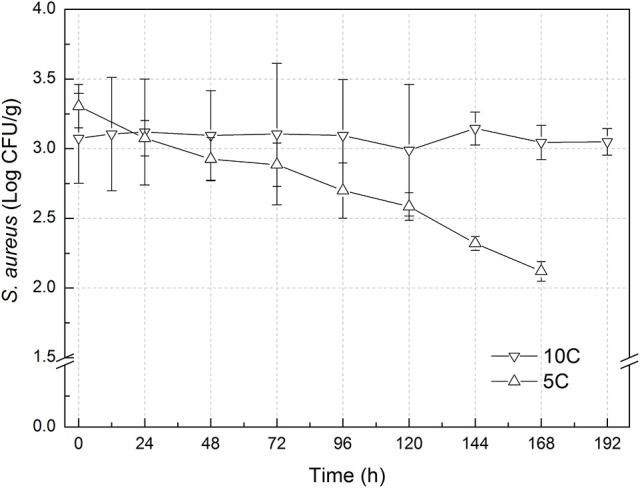
Observed growth data of *Staphylococcus aureus* on rice cake at 5 and 10°C.

The experimental data of *S. aureus* on rice at all the designed temperatures for model development and validation were collected and presented in Figures 1, 2, respectively. Experimental observations revealed *S. aureus* to be undetectable on the uninoculated rice cake samples (control). *S. aureus* did not grow well on the rice cake stored at 5 and 10°C (Figure 3), and a decline in the *S. aureus* cells was observed at 5°C. This was similar with previous studies which indicated *S. aureus* could grow at temperatures as low as 7°C (ICMSF, 1980). In contrast, no growth was observed at 10°C for the storage period of up to 7 days. The reason may be because the target samples were different food matrices, and Staphylococci are particularly sensitive to nutrient depletion. The data at these lower incubation temperatures (5 and 10°C) were not included in the development and validation of the model. The final cell populations of *S. aureus* at 15, 25, 35, and 45°C were 7.45, 7.55, 7.44, and 7.65 log CFU/mL, respectively, indicating that higher temperatures led to higher final cell populations. Among them, the higher values at 15 and 25°C are due to the higher initial population levels. Similar results were demonstrated in our previous research, in which the temperature was more influential on pathogen growth on the food matrix than humidity (Wang and Oh, 2012; Wang et al., 2012).

### Development of *S. aureus* growth models on rice cake

#### Development of primary models

In order to develop suitable primary models, which fit the growth data of *S. aureus* on rice cake, the growth curves of *S. aureus* at different environmental conditions were fitted into the Baranyi and Roberts model. The fitted curves at different temperatures were shown in Figures 1, 2. The results of analysis of the derived growth parameters including μ and LT indicated no significant differences between μ and LT values among different relative humidity (*p* < 0.05), regardless of the storage temperatures. Figures 1, 2 showed that lag phase could be only relevant for low temperatures. Therefore, the experimental data collected at the different relative humidity levels were combined to estimate specific growth rate of *S. aureus* on rice cake. The results of fitted parameters are summarized in Table 1.

**Table 1:** Growth parameters (μ_max_, specific growth rate and lag time) of *Staphylococcus aureus* on rice cake at different experimental conditions.

**Temperature (°C)**	**μ_max_ (1/h)**	**Std. Error**	**95% CI^a^**		**Lag time (h)**	**Std. Error**	**95% CI**		***R*^2^**
			**Lower**	**Upper**			**Lower**	**Upper**	
15°C	0.157	0.014	0.130	0.184	7.860	3.520	0.961	14.759	0.948
25°C	0.372	0.013	0.346	0.398	3.850	0.624	2.627	5.073	0.989
35°C	0.771	0.024	0.724	0.818	0.865	0.330	0.218	1.512	0.989
45°C	0.779	0.029	0.722	0.836	0.923	0.398	0.143	1.703	0.987
20°C	0.394	0.064	0.269	0.520	5.770	2.452	0.964	10.575	0.983
30°C	0.600	0.037	0.528	0.673	2.018	0.768	0.512	3.524	0.974
40°C	0.858	0.084	0.694	1.023	1.993	0.866	0.296	3.690	0.959

a *Confidence intervals of the parameters*.

#### Development of secondary models

Following the test of statistical significance of μ obtained from the primary models, only temperature was considered as a factor influencing the growth of *S. aureus* on rice cakes. The square root model, a special case of Belehradék's temperature function with the exponent of two, was suitable to describe the relationship between temperature and microbial growth rates (McMeekin et al., 2013). All μ-values estimated from the primary models were employed to develop the secondary models using a square root model as follows:

(11) $$\sqrt{\mu \;}=0.0173^{*}(\text{T}+10.03)$$

The fitted parameters of the Equation (11) are summarized in Table 2.

**Table 2:** Estimated Specific growth rate (μ_max_) of the secondary model for growth of *Staphylococcus aureus* on rice cake.

	**parameter**	**Value**	**L95CI^a^**	**U95CI**
GR	a	0.0173	0	0.034
	Tmin	−10.03	−51.5	31.4

a *Confidence intervals (95%) of the estimates*.

#### Validation of predictive models

In the present study, external validation was conducted using additional data under experimental conditions (Figure 2) which were not used for development of the models. Bias and accuracy factors are usually calculated for model validation. The B_f_ value of the predictive model for μ was 1.131. Based on the criterion proposed by Ross (1999), the results indicated that the performance of developed models could be considered as acceptable. The acceptable range of an accuracy factor determined the number of environmental parameters in a kinetic model with 0.10–0.15 units increased for each predictive variable (Ross et al., 2000). In this study, the acceptable range for A_f_ should be <1.15 since just temperature was employed. The A_f_ value of the predictive model for μ was 1.131. The results indicated that the performance of developed model for μ could be considered as acceptable. RMSE was used to evaluate the performance of the predictive model. The lower the RMSE, the better goodness-of-fit the model shows (Wang et al., 2012). The RMSE value of the predictive model for μ was 0.086. Previous works reported errors of the prediction of growth rate ranging from 0.27 to 0.30 (Sutherland et al., 1994; Olmez and Aran, 2005), which was greater than that for μ in the present study. The results indicated that the developed model showed a good quality of fit for the experimental data. The %SEP has the advantage of being dimensionless, and calculation of the %SEP for REs has the advantage of not being dependent on the magnitude of the measurements. The %SEP value of the predictive model for μ on rice cake was 11.1%. In several previous studies, it was reported that the %SEP value for growth rate of *Leuconostoc mesenteroides* for external validation in aerobic conditions ranged from 14.37 to 22.88% (Garcia-Gimeno et al., 2005; Hervas-Martinez et al., 2006). Compared with the previously reported results, both values obtained herein proved the acceptable goodness of the proposed models.

### *S. aureus* growth on rice cake at dynamic temperatures

**Figure 4:**
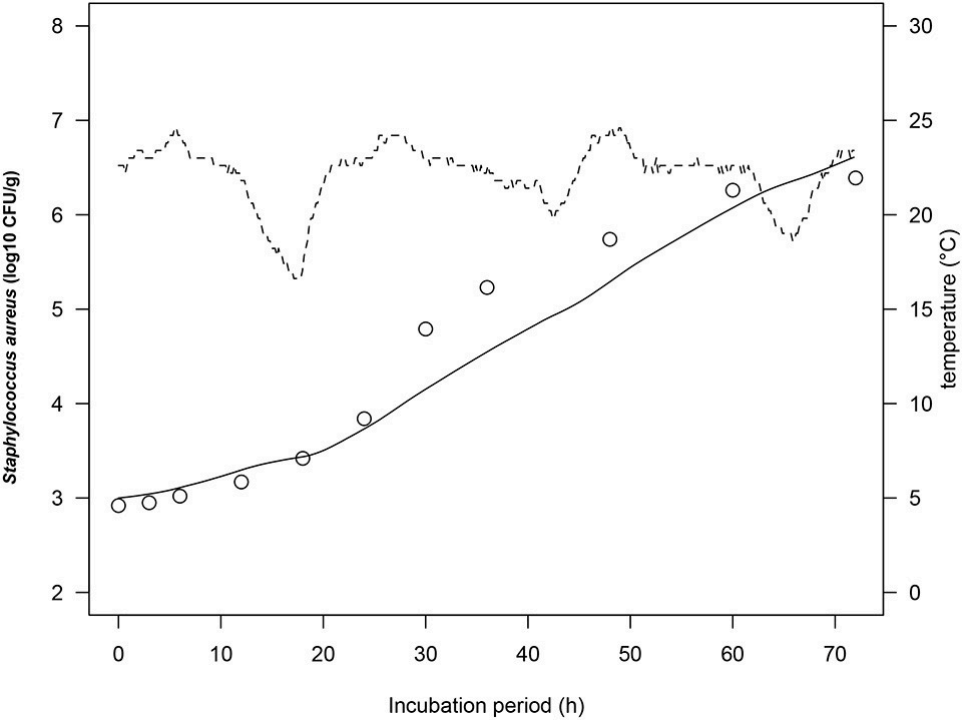
Growth curves of *Staphylococcus aureus* on rice cake at dynamic temperatures (dashed lines) during the stimulated storage period. The solid line represents the prediction made from the developed model for *S. aureus*. ○ represents the observed values of *S. aureus* growth on rice cake.

To simulate growth of *S. aureus* on rice cake under real temperature profile, we calculated the number of *S. aureus* by using simultaneous differential equations (Equations 2, 3). The physiological state parameter *q*_0_ was determined by average of the estimated fitted parameters of four iso-thermal growth curves. The *q*_0_ of the tested growth curves of *S. aureus* was determined as 0.20 ± 0.14. Figure 4 demonstrated one of the representing results under fluctuating temperature condition. The RMSE for *S. aureus* growth on rice cake was 0.218, while the acceptable prediction rate was 90.9%. These indices exhibited acceptable performance of the developed model for *S. aureus* growth on rice cake under the tested temperatures. In the present study, just one of the representing fluctuating temperature condition was monitored. The model was not tested for significant changes in temperature. Although the predictive capabilities of the model under dynamic temperature were not conducted, the calculation procedure would enable to correspond to real temperature history during distribution.

Fujikawa et al. (2004) developed a new logistic model for bacterial growth at dynamic temperatures using a numerical solution with the fourth-order Runge–Kutta method and demonstrated that the newly developed model could successfully predict *Escherichia coli* and *Salmonellae* growth curves for various patterns of the temperature history. Subsequently, *S. aureus* growth in sterilized milk were successfully predicted at various patterns of varying temperature using the newly developed logistic model, while its enterotoxin amounts predicted with the developed regression line for Staphylococcal enterotoxins were higher than the observed values (Fujikawa and Morozumi, 2006). In addition, comparison of the dynamic models based on the Baranyi model and modified logistic model could produce almost the same growth curves for varying temperature histories (Fujikawa and Morozumi, 2005).

### Development of probabilistic model

In order to determine the effect of temperature on the growth of *S. aureus* on rice cake during the storage period, the probability of the time required to reach a certain level was determined using the logistic regression model described in Equation (4). The estimated parameters of the logistic regression models for 1-, 2- 3-, and 4-log increases of *S. aureus* expansion on rice cake are presented in Table 3. The information of goodness-of-fit of the model, such as 95% confidence intervals of the parameters, maximum rescaled R^2^, area under ROC curve, and Hosmer-Lemeshow goodness of fit are also listed in Table 3. According to the performance statistics, the derived parameters for the developed logistic regression models showed excellent discrimination for all the log increases.

**Table 3:** Estimated parameters of the logistic regression for arbitrary growth increases of *Staphylococcus aureus* on rice cake.

**Increases**	**Parameters**	**Estimates**	**Std. Error**	**95% CI^a^**		**Maximum rescaled *R*^2^**	***c* (area under ROC^b^ curve)**	**Hosmer-lemeshow (goodness of fit)**
				**Lower**	**Upper**			
1-log	α0	−22.317	3.950	−30.058	−14.576	0.806	0.974	2.938 with 8 df^c^ (*P* = 0.938)
	α1	0.355	0.067	0.225	0.486			
	α2	5.835	1.002	3.872	7.799			
2-log	α0	−32.48	5.542	−43.342	−21.618	0.831	0.981	4.492 with 8 df (*P* = 0.810)
	α1	0.441	0.079	0.286	0.596			
	α2	7.667	1.288	5.143	10.191			
3-log	α0	−33.43	5.236	−43.693	−23.167	0.785	0.970	2.688 with 8 df (*P* = 0.952)
	α1	0.435	0.072	0.294	0.576			
	α2	7.077	1.092	4.937	9.217			
4-log	α0	−29.500	5.064	−39.425	−19.575	0.567	0.942	8.931 with 8 df (*P* = 0.348)
	α1	0.400	0.070	0.263	0.537			
	α2	4.998	0.898	3.238	6.758			

a *Confidence intervals (95%) of the estimates*.

b *Receiver operating curve*.

c *Degree of freedom*.

**Figure 5:**
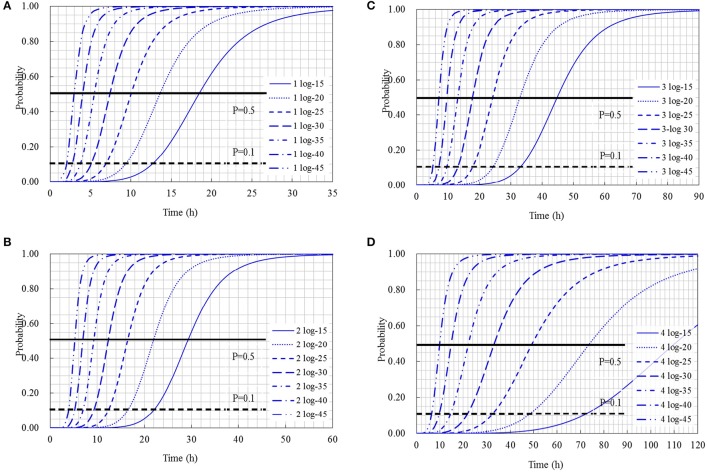
Probability of a certain log increases of *Staphylococcus aureus* occurring at different temperatures: **(A)** 1-log increases; **(B)** 2-log increases; **(C)** 3-log increases; **(D)** 4-log increases. Solid and dashed lines represent the probability (*p* = 0.5 and *p* = 0.1) reaching to a certain log increases of *S. aureus* growth.

**Figure 6:**
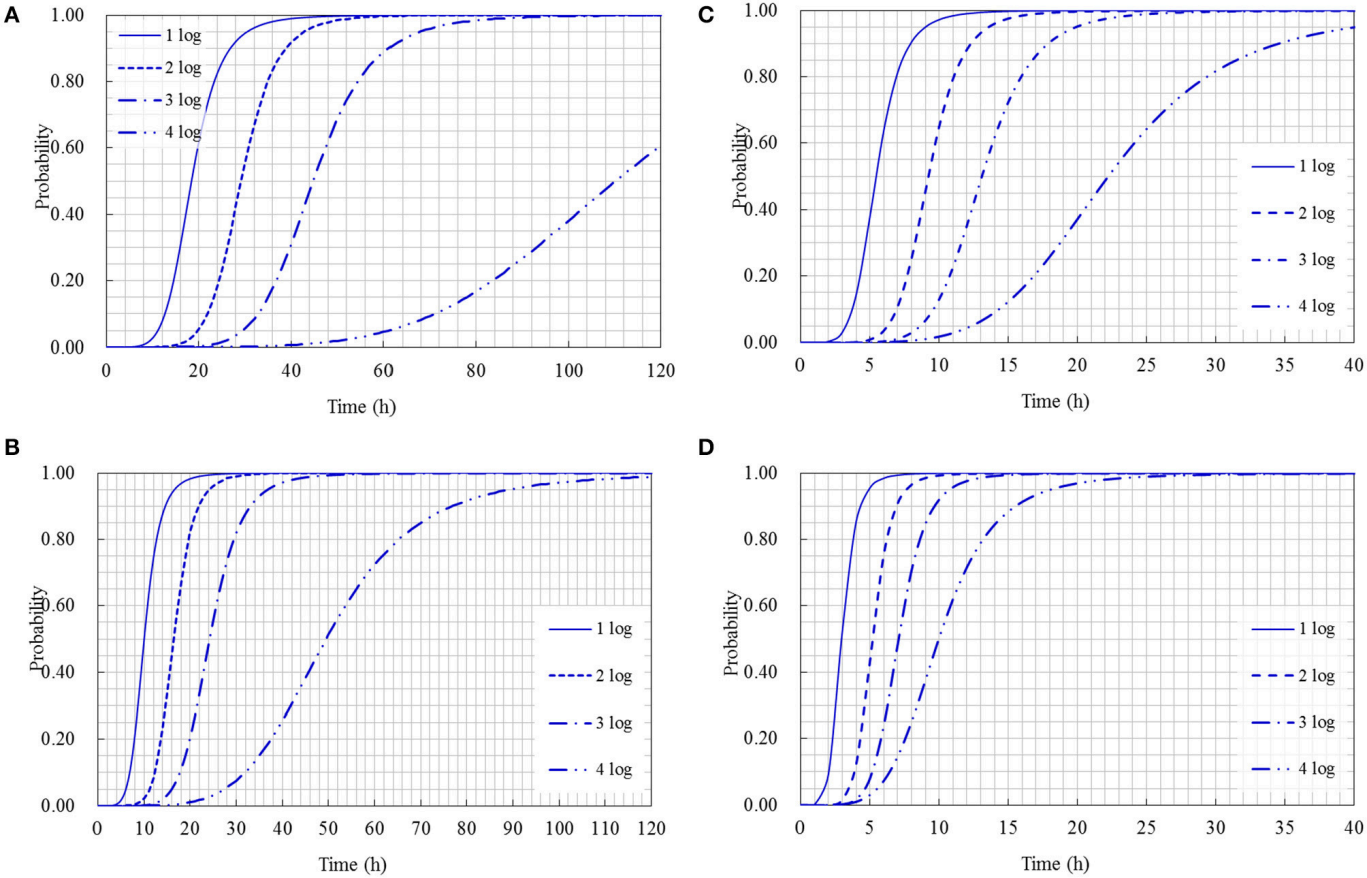
Probability of different log increases of *Staphylococcus aureus* at a certain time at the following temperature: **(A)** 15°C; **(B)** 25°C; **(C)** 35°C; **(D)** 45°C.

The cumulative probability distributions predicted by the developed models listed in Table 3 are shown in Figure 5 for the 1-, 2-, 3-, and 4-log increases of *S. aureus* on rice cake, respectively. From the figure, the probability of the time required to reach a certain level of *S. aureus* contamination on the rice cakes at different temperatures could be determined. Meanwhile, the probability of the time required to reach different levels of *S. aureus* on rice cake at a certain temperature is presented in Figure 6.

Using the obtained model, the Logit (P) values can be obtained, and the probabilities of *S. aureus* growth on rice cake can be estimated. Referred the reported limits on the growth of bacteria (Presser et al., 1998; Tienungoon et al., 2000; Le Marc et al., 2005; Hwang and Juneja, 2011) that *p* ≤ 0.1 indicates an “unlikely to grow” or “no-growth” region, *p* > 0.5 indicates a “likely to grow” or “growth” region, and p values between 0.1 and 0.5 indicate an “uncertainty” region, in the present study, the criterion of a certain log increases of *S. aureus* was set to be *P* = 0.5 and *P* = 0.1. That is to say, as for a certain level of logistic regression model, *P* ≤ 0.1 indicates an “unlikely reach” region of a certain log increase, *P* > 0.5 indicates a “reach” region, and *P*-values between 0.1 and 0.5 indicate a “likely reach” region (Figure 5). According to this criterion, we could derive an equation through transformation from the obtained logistic regression models shown in Table 3 to calculate the time to reach a certain log increase of *S. aureus* at different temperatures within the limits of the experimental design. The equations to calculate how long time to reach 1-, 2-, 3-, and 4-log increase of *S. aureus* were listed as following:

(12) t=exp ((22.317−0.355∗Temp)/5.835)

(13) t=exp ((32.48−0.441∗Temp)/7.667)

(14) t=exp ((33.43−0.435∗Temp)/7.077)

(15) t=exp ((29.50−0.400∗Temp)/4.998)

where Temp is the storage temperature, and t is the time at which 1-, 2-, 3-, and 4-log increases of *S. aureus* reached, respectively.

Time and temperature abuse would lead to results of pathogenic bacteria growth and toxin formation. *S. aureus* concentrations of more than 10^5^ CFU/mL were unacceptable, since numbers of *S. aureus* higher than 10^5^ most often lead to human illness (Food and Drug Administration, 2012). In addition, it is extremely likely to produce Staphylococcal enterotoxins under specific environmental conditions when the *S. aureus* level reaches 10^5^ CFU/mL (Heidinger et al., 2009). Fujikawa and Morozumi (2006) reported that temperature dependency of the rate constant of staphylococcal enterotoxin A (ng/mL/h) could be calculated as follows:

(16) $${\text{p}}_{\text{toxin}}=0.0376*T-0.559$$

where p_toxin_ is the production rate of staphylococcal enterotoxin A, T is temperature. Based on Equation (16), enterotoxin production rate at each temperature would be estimated. According to Equation (14), it is easy to know the time to reach 3-log increase of *S. aureus* growth at different temperatures within the limits of the experimental design. As shown in Figure 5c, the area above the solid line was “reach” region, and the time to reach 3-log increase of *S. aureus* growth at 15, 25, 35, and 45°C were 44.8, 24.2, 13.1, and 7.1 h, respectively. At the same time, the probability to reach 3-log increase could be obtained. Finally, the hazardous levels at each temperature could be evaluated considering the probability models and the enterotoxin production rate. If we could check the initial contamination level of rice cake, it is convenient to monitor the status of rice cake products. These logistic regression models could identify the shelf life of rice cake based on the initial levels of *S. aureus* contamination. From a food industry standpoint, to control the growth conditions of pathogens contributing to levels exceeding 10^5^ will be of key importance. Therefore, the developed probability models will be very useful for food safety management and microbiological risk assessment of *S. aureus* on rice cake in Korea.

## Author contributions

The experiments were conceived and designed by JW, MC, and DO. Microbiological and data analyses were performed by JW and SK. The paper was written by JW, SK, and DO.

### Conflict of interest statement

The authors declare that the research was conducted in the absence of any commercial or financial relationships that could be construed as a potential conflict of interest.
